# Supplementary material for: Haplotype mapping uncovers unexplored variation in wild and domesticated soybean at the major protein locus cqProt-003
Source: Theor Appl Genet. 2022 Feb 9;135(4):1443–55. doi: 10.1007/s00122-022-04045-8 (PMC9033719; doi:10.1007/s00122-022-04045-8)
Supplement: Supplementary file 2 — Supplementary file2 (DOCX 1828 kb) [file 122_2022_4045_MOESM2_ESM.docx]

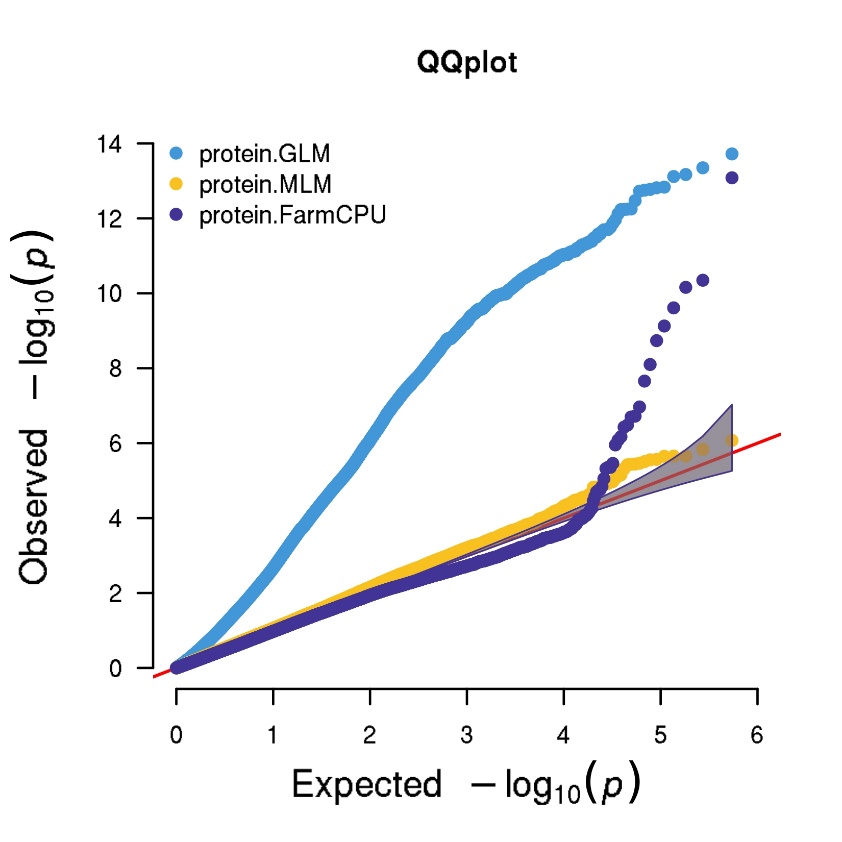


**Figure S1.** **Multitrait QQ plots of GLM, MLM and FarmCPU for protein GWAS using unimputed SNPs** filtered only for quality. Each dot represents a SNP. To support GWAS results the vast majority of markers should be uncorrelated with the phenotype and adhere to the red line; conversely some markers correlated with the phenotype of interest should possess more significant Observed than Expected p-values (a tail skewed up).


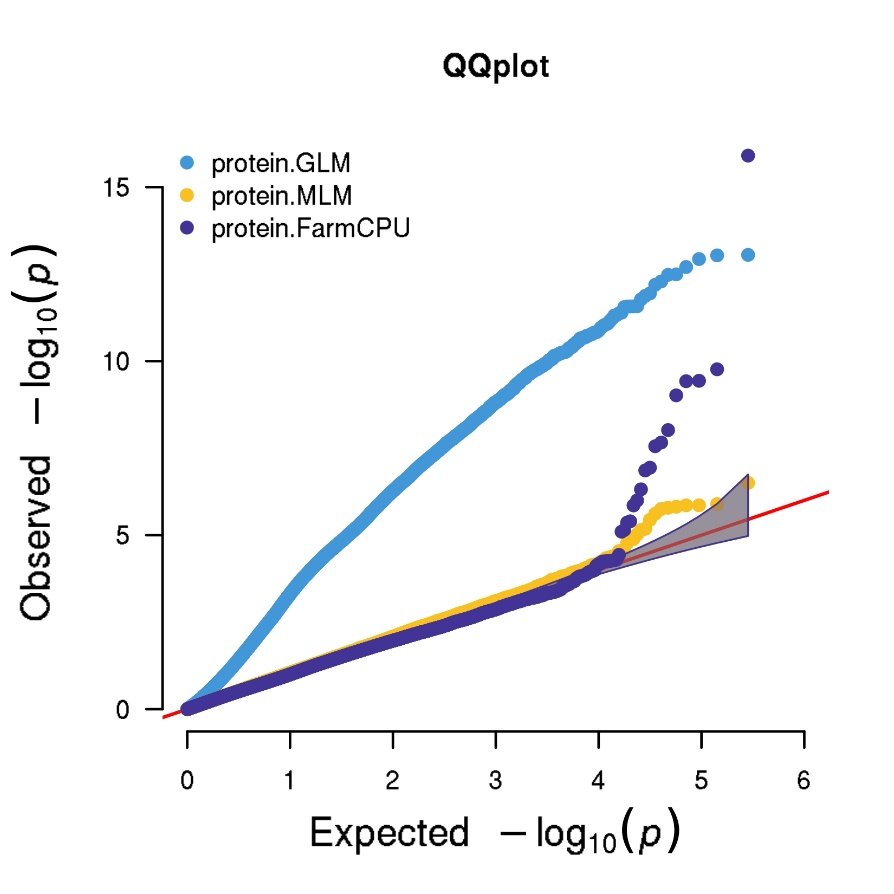


**Figure S2.** **Multitrait QQ plots of GLM, MLM and FarmCPU for protein GWAS using unimputed SNPs and reformatted InDels** filtered for minor allele frequency and missingness. Each dot represents a SNP. To support GWAS results the vast majority of markers should be uncorrelated with the phenotype and adhere to the red line; conversely some markers correlated with the phenotype of interest should possess more significant Observed than Expected p-values (a tail skewed up).


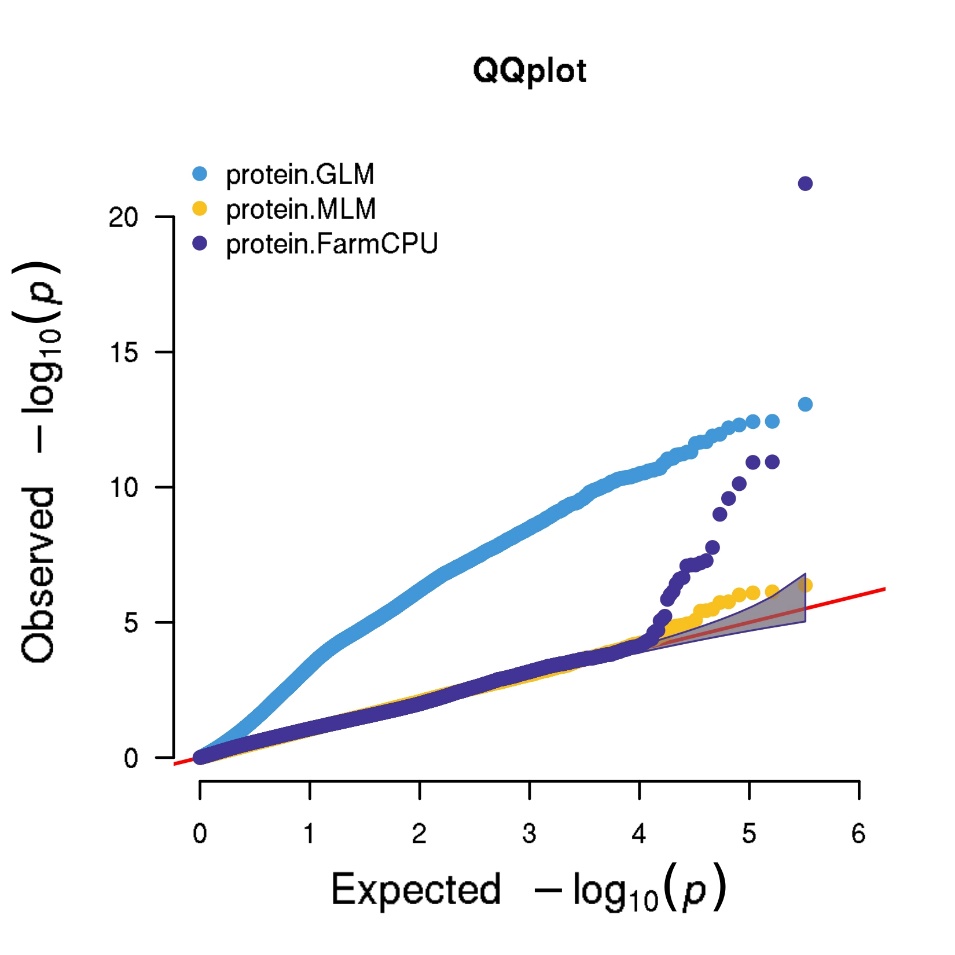


**Figure S3.** **Multitrait QQ plots of GLM, MLM and FarmCPU for protein GWAS using imputed SNPs** filtered for minor allele frequency and missingness. Each dot represents a SNP. To support GWAS results the vast majority of markers should be uncorrelated with the phenotype and adhere to the red line; conversely some markers correlated with the phenotype of interest should possess more significant Observed than Expected p-values (a tail skewed up).


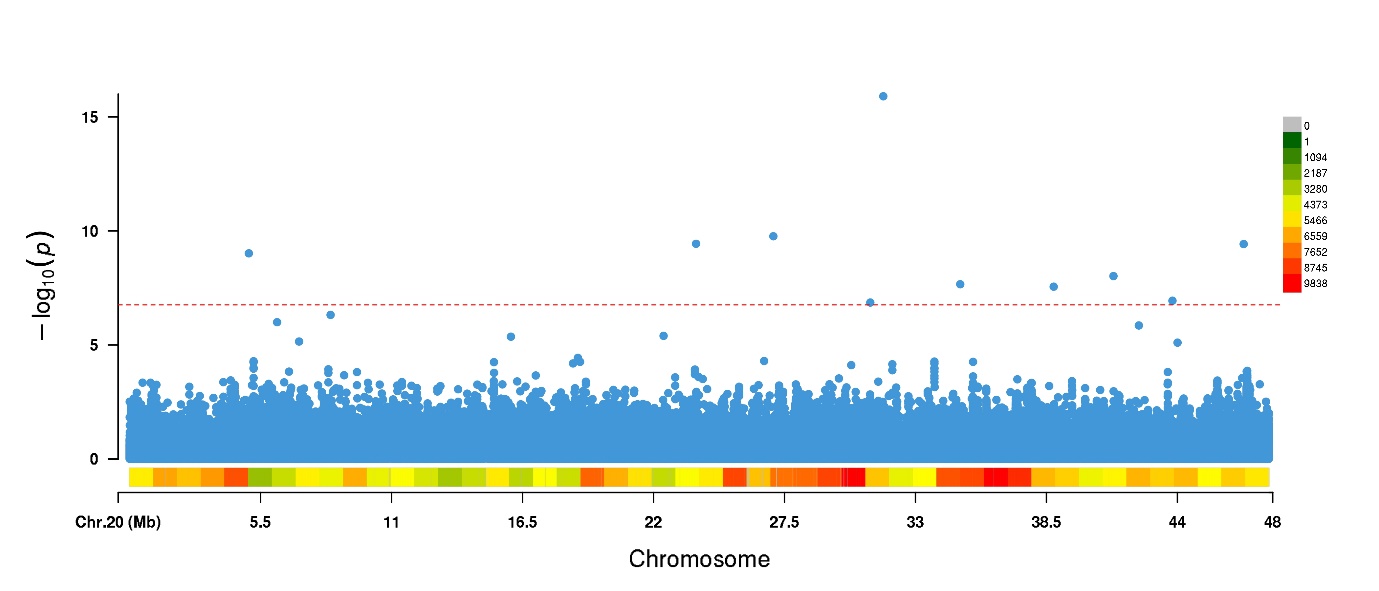


**Figure S4.** **Manhattan plot for protein FarmCPU GWAS** on chromosome 20 using unimputed SNPs filtered for minor allele frequency and missingness. Each point is a SNP; the red dashed line indicates the –E^10^ p-value significance threshold.


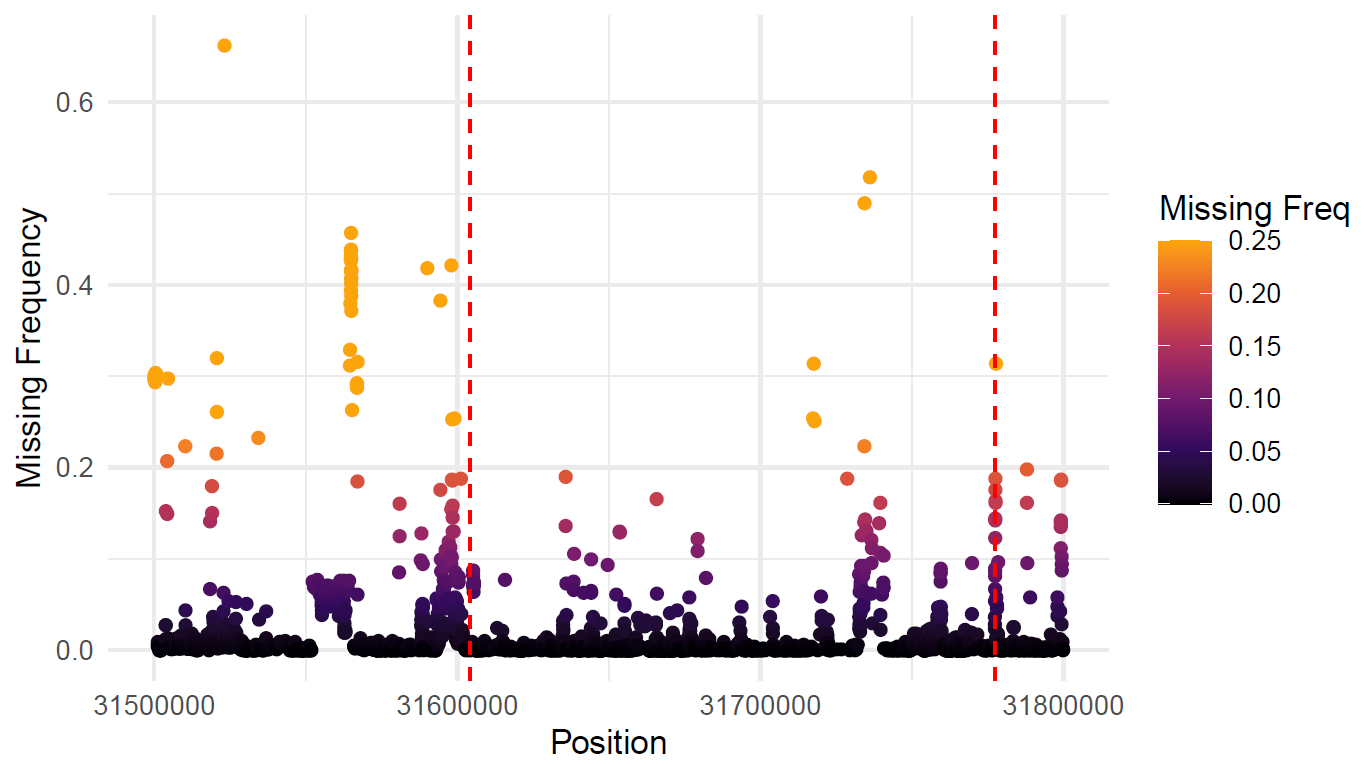


**Figure S5. Frequency of missingness by site around cqProt-003 region.** Dashed red lines delimit the 173kb region defined in this study. Each dot represents a SNP locus filtered to minor allele frequency > 0.01.


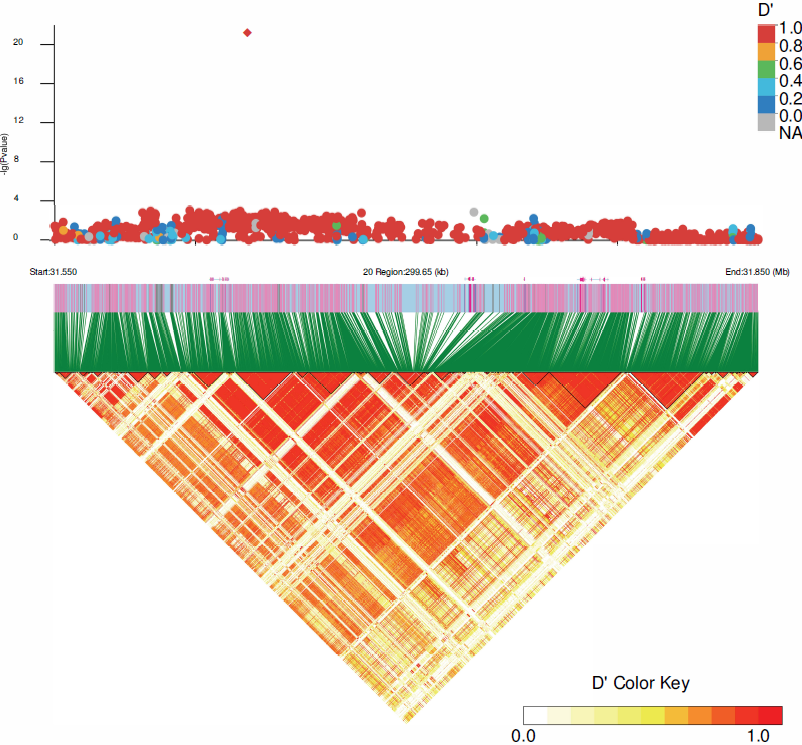


**Figure S6. Linkage disequilibrium of D' between SNPs across a 300Kb region surrounding the significant GWAS-SNP**. The red diamond in the dot plot above indicates the GWAS-SNP, with the colour of the other dots representing D' linkage values with the GWAS-SNP in discrete increments. The heat map below indicates linkage of all SNPs in the region with eachother with thin black lines delineating linkage blocks as defined through the confidence interval approach (Purcell et al. 2007).


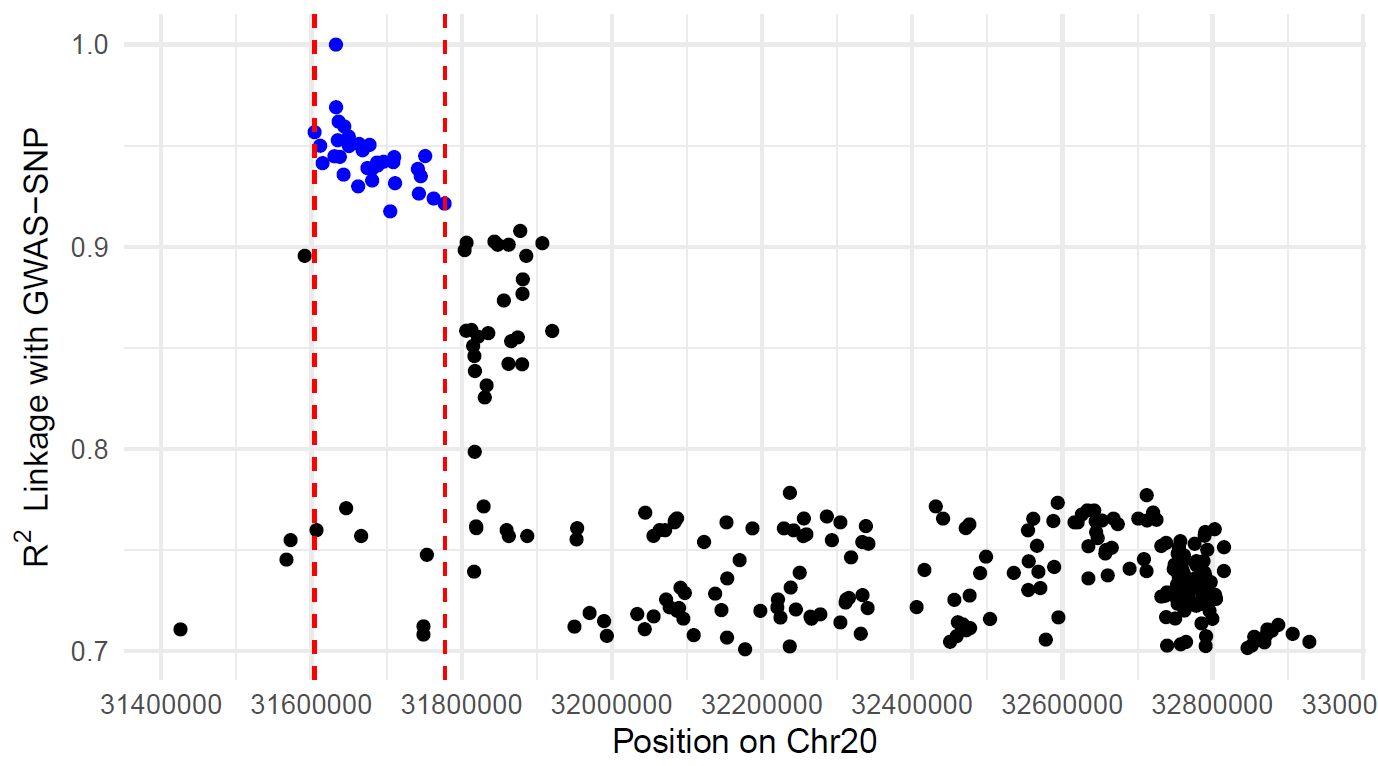


**Figure S7. Linkage (R^2^) of small variants with GWAS-SNP,** by position. Blue points indicate filtered SNP variants included in both the M01 marker group (Table S5) and final high protein haplotype (Table S9), dashed red lines delimit the 173kb region. N.B. markers in M01 extending downstream of the 173kb region are not coloured.


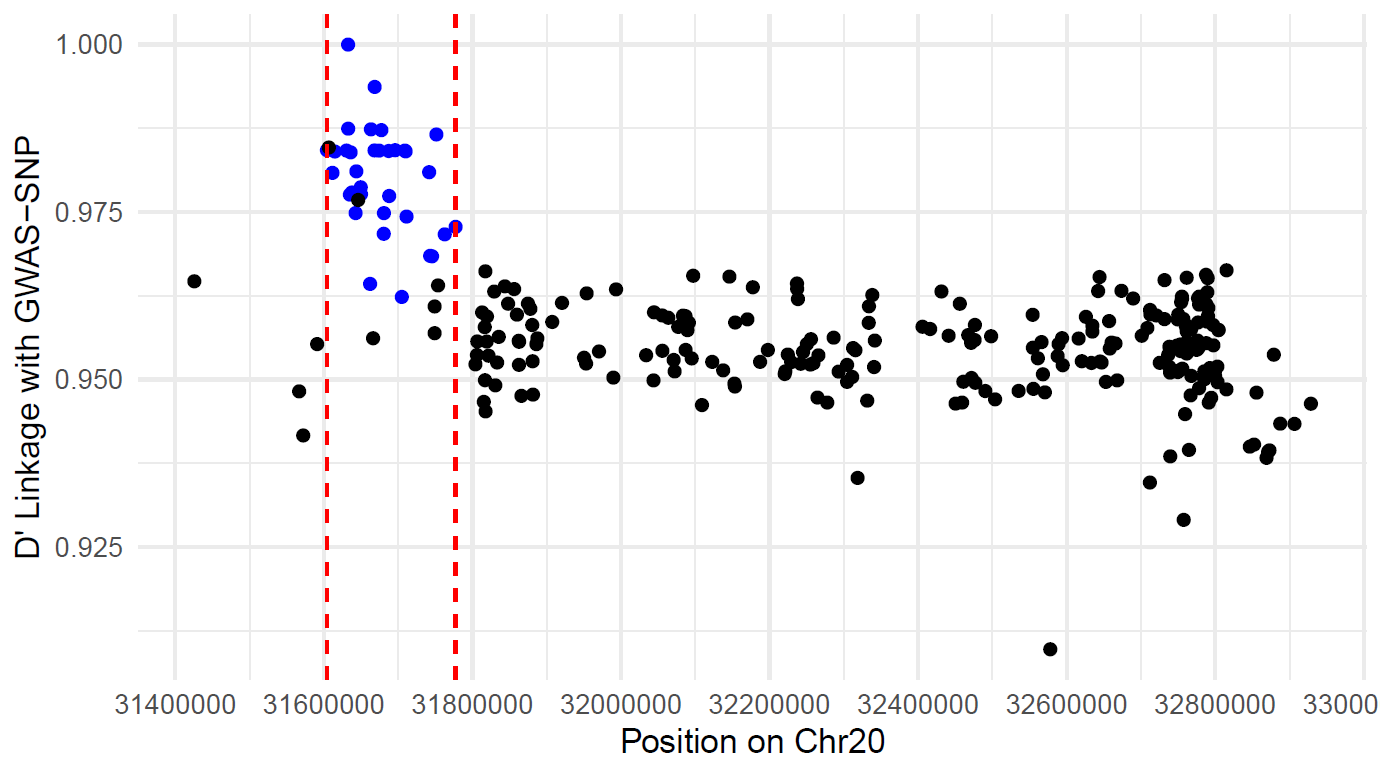


**Figure S8. Linkage (D')** **of small variants with GWAS-SNP,** by position. Blue points indicate filtered SNP variants included in both the M01 marker group (Table S5) and final high protein haplotype (Table S9), dashed red lines delimit the 173kb region. N.B. markers in M01 extending downstream of the 173kb region are not coloured.


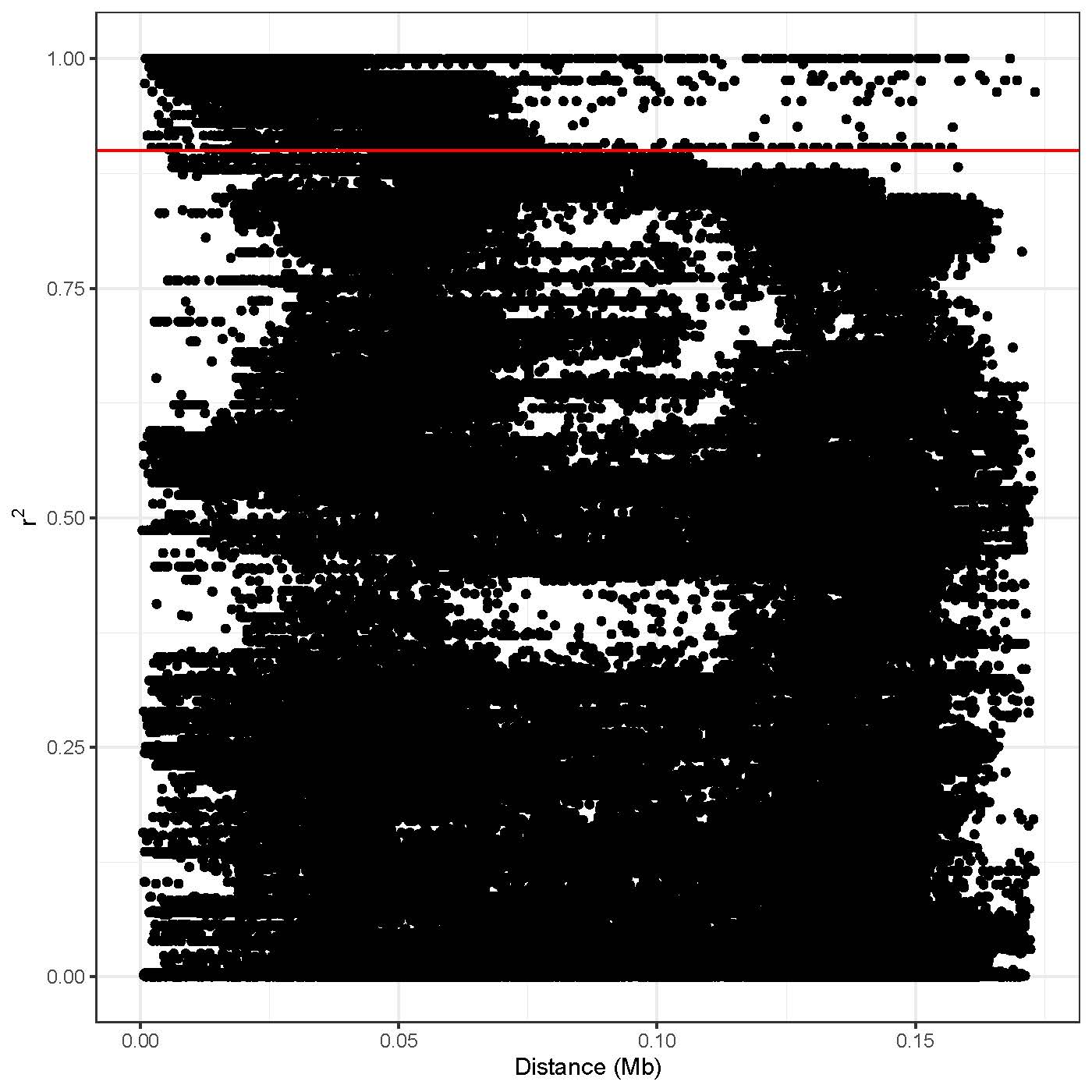


**Figure S9. Estimation of linkage disequilibrium (R^2^) between all pairs of markers across the 173kb region as a function of pairwise distance,** taken directly from HaplotypeMiner (Tardivel et al. 2018) output**.** Markers above the horizontal red line were included in the gene-centric haplotyping analysis on the basis that they exhibit very high linkage (R^2^>0.9).


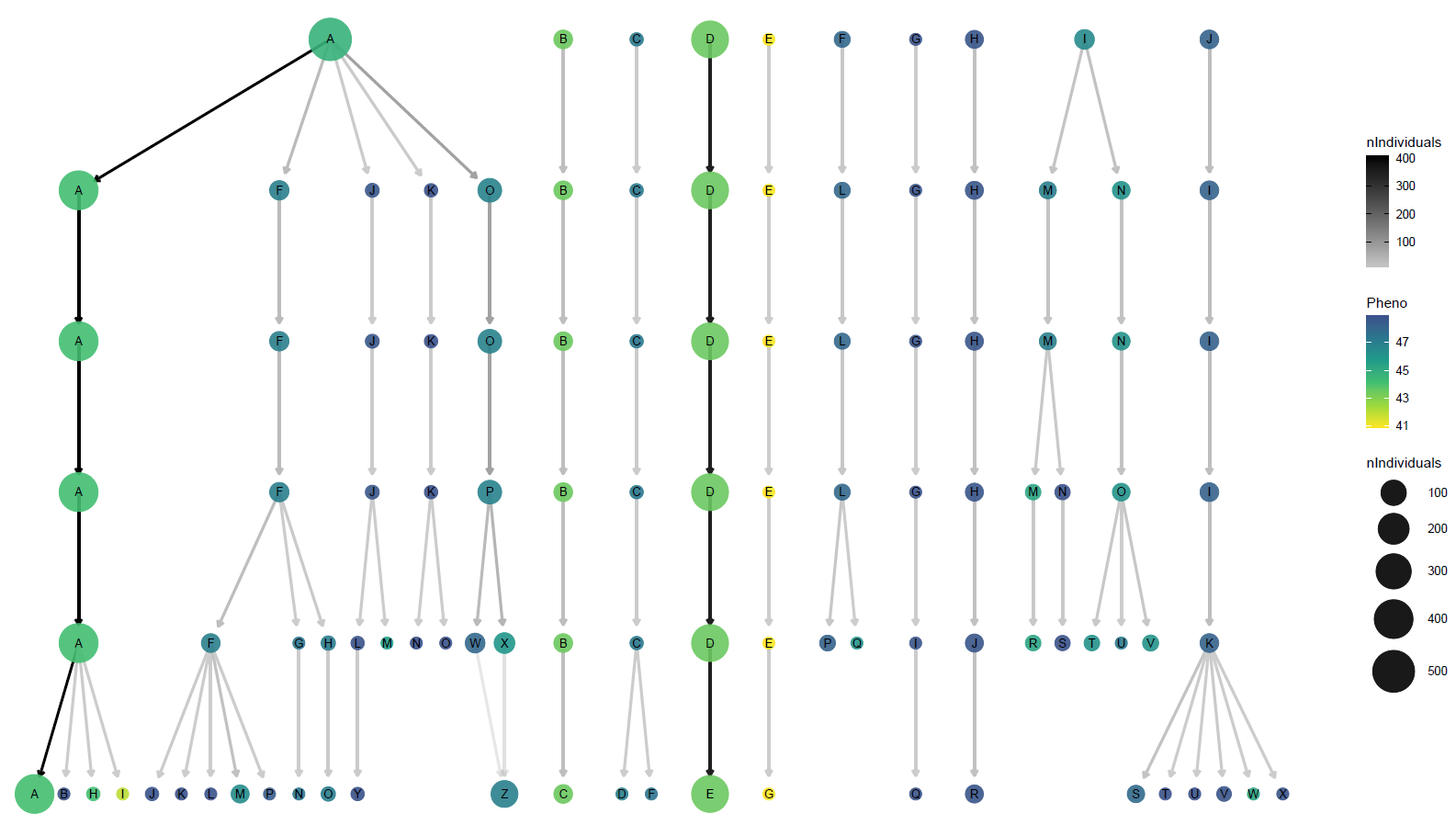


**Figure S10: Stability of clusters of individuals who share haplotype combinations at different cluster thresholds (CT)**. CT= 0.5 at the top, each lower row represents a decrease in CT of 0.1, with CT = 1.0 at the bottom; the chosen CT of 0.6 is highlighted by the red circle. An appropriate clustering threshold will retain cluster stability across small changes in clustering threshold. N.B. this includes clusters removed due to redundancy, thus the letters for haplotype populations do directly correspond to those reported in the manuscript.


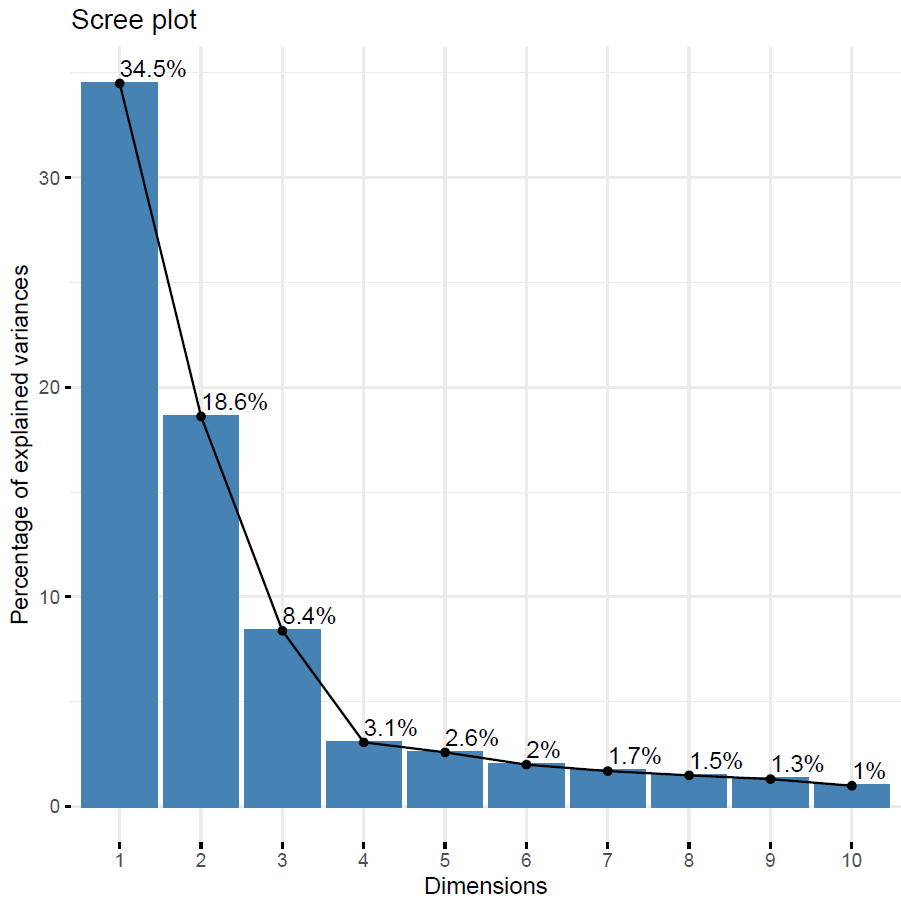


**Figure S11: Scree plot displaying proportion of total genetic variance between individuals explained by principal components.** PCA was run across individuals using SNPs from within the 173kb region only. The chosen number of dimensions for input to the UMAP analysis was the top 7. This means a cumulative total of 70.9% of the total variance between individuals is explained in Figure S12.


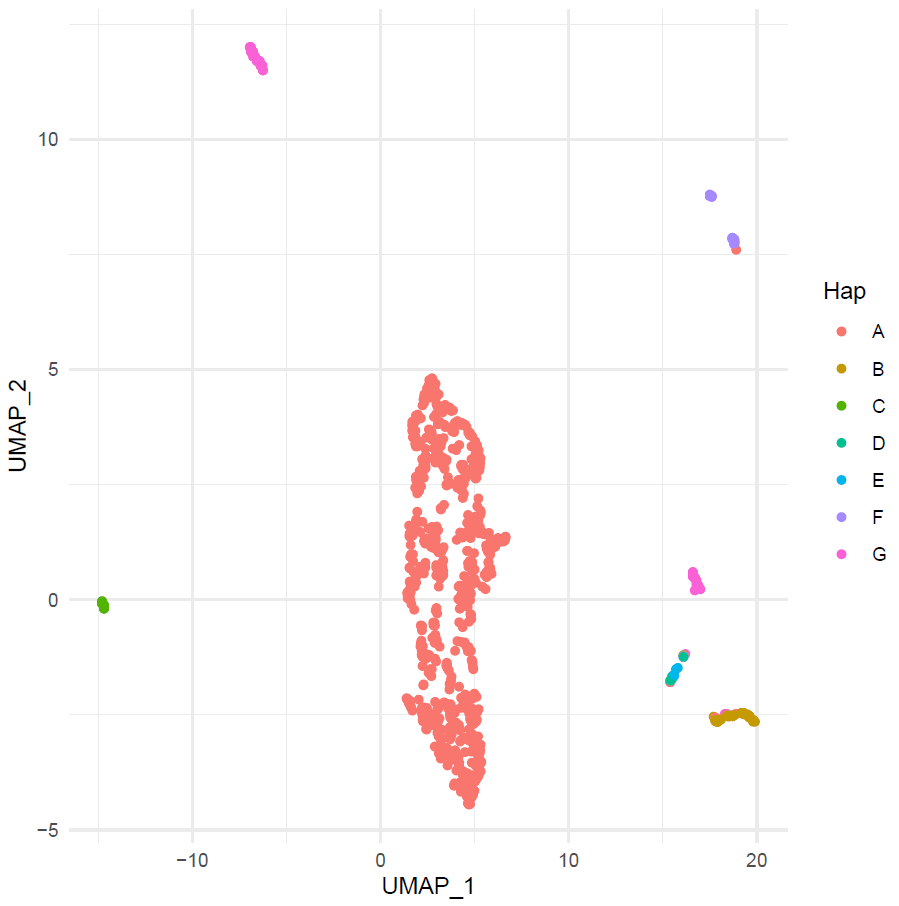


**Figure S12: Dimensionally reduced projection (UMAP) for assessing segmentation between haplotype groups (A-G).** Each dot represents an individual, clustered based on genetic similarity in the 173kb region. The Hap A individuals that are not located in the central cluster can be explained as being 6 atypical A haplotype individuals (Table S6): HN039 is wild and heterozygous at GWAS-SNP; HN092 and SRR1533182 are heterozygous for trinucleotide insertions at 31,727,019 bp; PI507638 is wild and has the 304bp deletion at 31,728,619 bp; PI378696B and PI378696A are wild and contain homozygous trinucleotide insertions at 31,727,019 bp.


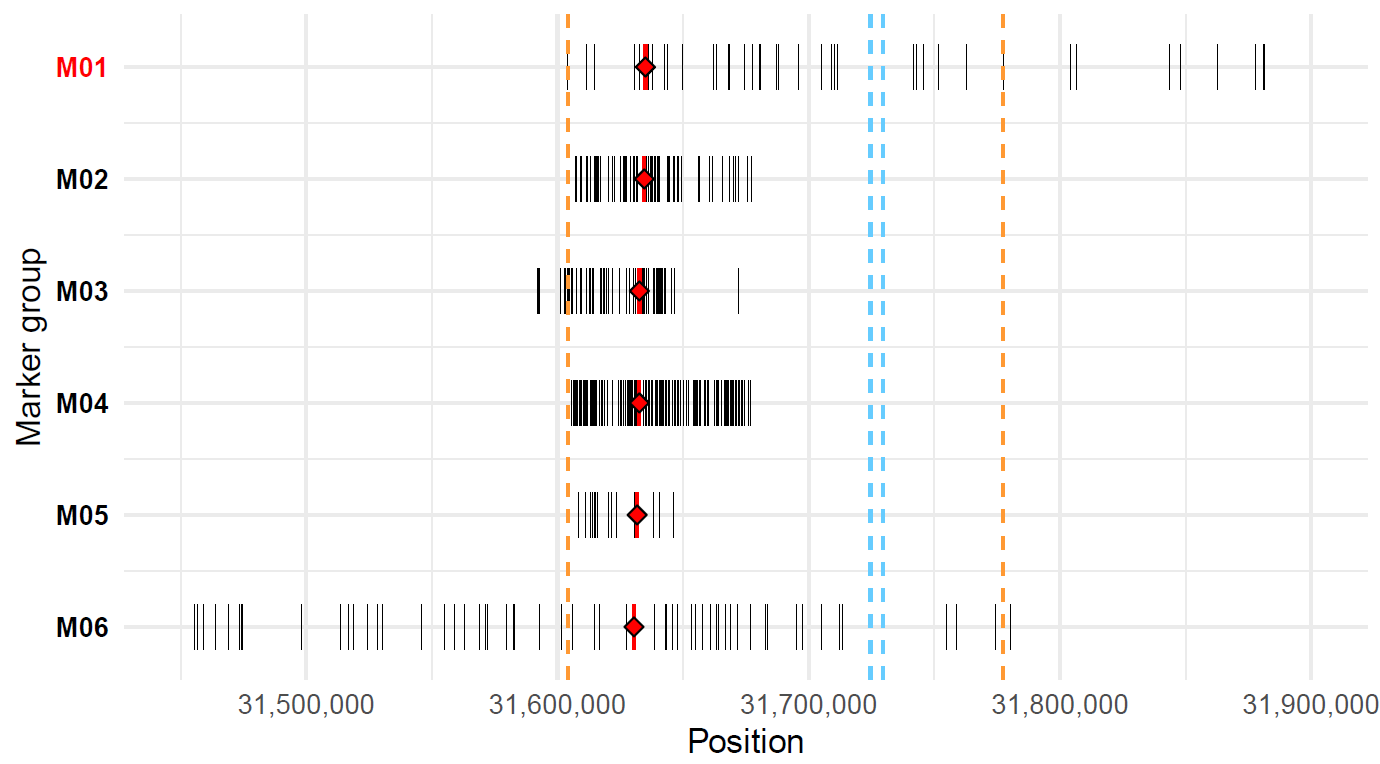


**Figure S13. Genomic position on Chromosome 20 of representative and supporting markers contained within each marker group (M01-M06)** using filtered, unimputed SNPs (Table S5). The representative markers cluster around 31,633,200 because it is the centre of the PLINK linkage block containing the GWAS-SNP, which was chosen as the “gene” region input to HaplotypeMiner. Supporting SNPs were defined by linkage (R^2^>0.9) with the representative marker. Each vertical line represents a single locus, representative sites are marked red with a diamond. The red label is for the marker group of interest that is most associated with protein content (M01). The blue vertical dashed line delimits the start and end of the *Glyma.20G085100* gene. The orange vertical dashed line delimits the start and end of the 173kb region which contains SNPs in high linkage with the GWAS-SNP. N.B. Eight SNPs supporting M01 extend downstream of the 173kb region, yet aren’t reported in the high protein haplotype (Table S9), this is because they are in higher linkage with the M01 representative site (31634985) than with the GWAS-SNP (31,632,556)
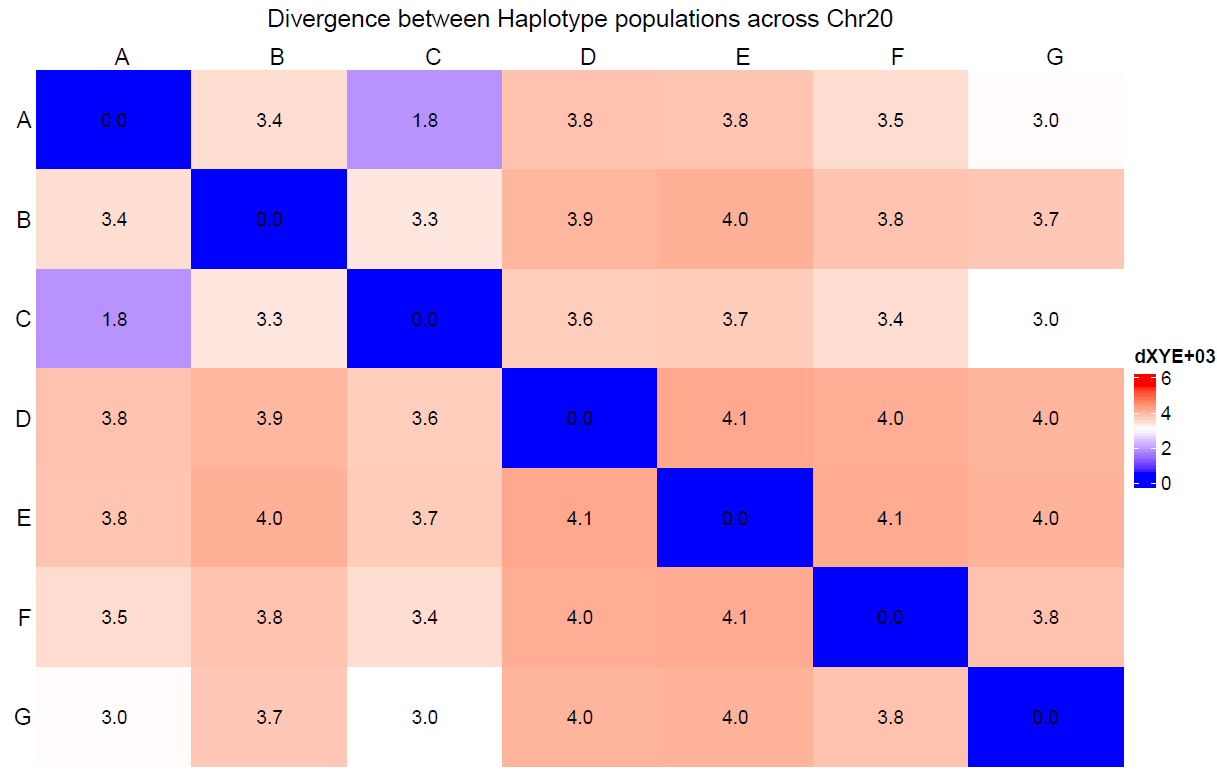


**Figure S14. Whole chromosome general divergence (dXY) between haplotype group populations** using filtered, unimputed SNPs including invariant sites to avoid over-estimation. Red cells indicate the two corresponding haplotype groups (see X-axis and Y-axis labels) are genetically more divergent across the entirety of chromosome 20, blue indicates similarity, with white as a midpoint.


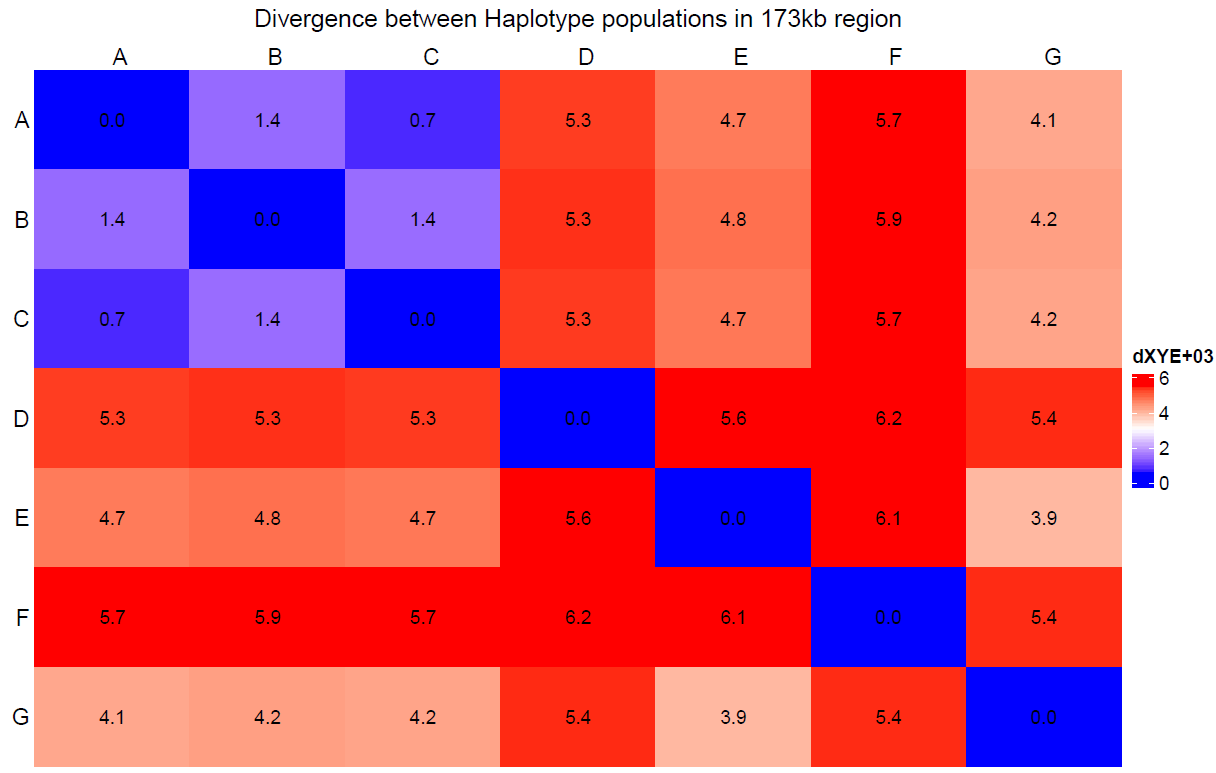


**Figure S15. 173kb region specific divergence (dXY) between haplotype group populations** using filtered, unimputed SNPs including invariant sites to avoid over-estimation. Red cells indicate the two corresponding haplotype groups (see X-axis and Y-axis labels) are genetically more divergent within the 173kb region, blue indicates similarity, with white as a midpoint.


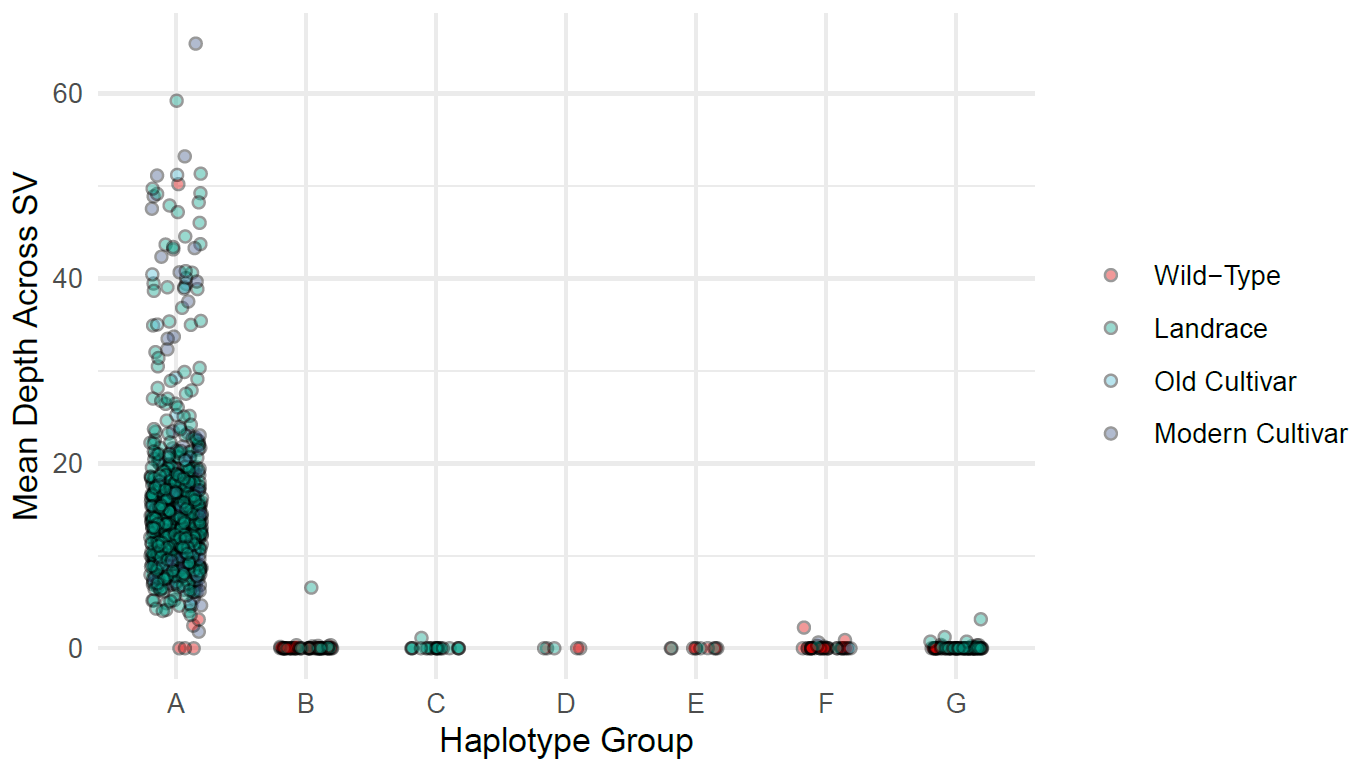


**Figure S16. Mean sequencing read depth calculated using Mosdepth across 304bp SV from 31,728,619 to 31,728,923 for individuals, grouped by haplotype.** Using sequence alignments the Wm82.a4.v1 reference. Mean depth at or very near 0 is evidence for the 304bp deletion seen in many high-protein individuals, whereas higher mean depth is evidence for its presence as seen in many low-protein individuals.
